# A targeted door-to-door strategy for sleeping sickness detection in low-prevalence settings in Côte d’Ivoire

**DOI:** 10.1051/parasite/2016059

**Published:** 2016-11-16

**Authors:** Mathurin Koffi, Martial N’Djetchi, Hamidou Ilboudo, Dramane Kaba, Bamoro Coulibaly, Emmanuel N’Gouan, Lingué Kouakou, Bruno Bucheton, Philippe Solano, Fabrice Courtin, Stephan Ehrhardt, Vincent Jamonneau

**Affiliations:** 1 Université Jean Lorougnon Guédé, UFR Environnement, Laboratoire des Interactions Hôte-Microorganisme-Environnement et Évolution (LIHME) BP 150 Daloa Côte d’Ivoire; 2 Institut de Recherche pour le Développement (IRD), Unité Mixte de Recherche IRD-CIRAD 177, INTERTRYP /Centre International de Recherche-Développement sur l’Élevage en zone Subhumide (CIRDES), Unité de recherches sur les bases biologiques de la lutte intégrée 01 BP 454 Bobo-Dioulasso 01 Burkina Faso; 3 Institut Pierre Richet, Unité de Recherche « Trypanosomoses » 01 BP 1500 Bouaké 01 Bouaké Côte d’Ivoire; 4 Projet de Recherche Clinique sur les Trypanosomoses (PRCT) BP 1425 Daloa Côte d’Ivoire; 5 Programme National d’Élimination de la Trypanosomose Humaine Africaine 17 BP 934 Abidjan Côte d’Ivoire; 6 Institut de Recherche pour le Développement (IRD), Unité Mixte de Recherche IRD-CIRAD 177, INTERTRYP, Campus International de Baillarguet 34398 Montpellier Cedex 5 France; 7 Department of Epidemiology, Johns Hopkins Bloomberg School of Public Health, Baltimore Maryland United States of America

**Keywords:** Human African trypanosomiasis, *Trypanosoma brucei gambiense*, Diagnosis, Elimination strategy, Côte d’Ivoire, Medical survey

## Abstract

Significant efforts to control human African trypanosomiasis (HAT) over the three past decades have resulted in drastic reductions of disease prevalence in Côte d’Ivoire. In this context, the costly and labor-intensive active mass screening strategy is no longer efficient. In addition to a more cost-effective passive surveillance system being implemented in this low-prevalence context, our aim was to develop an alternative targeted active screening strategy. In 2012, we carried out a targeted door-to-door (TDD) survey focused on the immediate vicinities of former HAT patients detected in the HAT focus of Bonon and compared the results to those obtained during classical active mass screening (AMS) surveys conducted from 2000 to 2012 in the same area. The TDD that provides a friendlier environment, inviting inhabitants to participate and gain awareness of the disease, detected significantly more HAT cases than the AMS. These results suggest that the TDD is an efficient and useful strategy in low-prevalence settings where very localized transmission cycles may persist and, in combination with passive surveillance, could help in eliminating HAT.

## Abstract (fr)

D’importants efforts pour contrôler la trypanosomiase humaine africaine (THA) au cours des trois dernières décennies ont permis une réduction importante des prévalences en Côte d’Ivoire. Dans ce contexte, les prospections médicales classiques de masse ne sont plus adaptées en termes de coût-efficacité. En plus de développer un système de surveillance passive plus rentable dans ce contexte, notre but était de soutenir l’élimination de la THA en élaborant une stratégie active alternative basée sur une détection ciblée de cas. Dans le foyer de Bonon, nous avons mené en 2012 une surveillance médicale en porte-à-porte ciblée sur le voisinage immédiat d’anciens cas de THA et comparé les résultats à ceux des prospections médicales classiques de masse menées entre 2000 et 2012 dans la même zone. La stratégie ciblée en porte-à-porte, qui contribue à une meilleure sensibilisation des populations, a détecté significativement plus de cas de THA que la prospection médicale classique de masse. Ceci montre que la stratégie ciblée en porte-à-porte est une stratégie efficace dans les contextes de faibles prévalences où des cycles de transmission très localisés peuvent persister, qui est complémentaire à la surveillance passive pour atteindre l’objectif d’élimination de la trypanosomiase humaine africaine.

## Introduction

Human African trypanosomiasis (HAT), commonly known as sleeping sickness, is a debilitating and deadly disease that affects people in sub-Saharan Africa, mainly in rural areas [2, 7]. There is no vaccine and no chemoprophylaxis available. Significant efforts to control the disease over the past three decades have drastically reduced the prevalence of HAT in recent years [22]. The elimination of the disease as a public health problem by 2020 is now regarded as an achievable goal and has been included in the WHO roadmap on neglected tropical diseases [8, 22, 26]. However, one condition of success is the use of the most sensitive and specific diagnostic tools and the most cost-effective strategies, taking into account local epidemiological contexts and the current decrease in prevalence [8, 26].

The chronic form of HAT is assumed to be mainly an anthroponosis due to *Trypanosoma brucei gambiense*, today causing more than 95% of all reported cases in West and Central Africa [7]. Control is mainly based on active case detection and treatment of the human reservoir of the parasites [26]. Such active case detection, mainly based on the Card Agglutination Test for Trypanosomiasis (CATT) serological test [19] followed by parasitological investigation, is usually done by mobile teams that move from village to village to examine the entire population in the HAT foci aiming to detect and treat cases as early as possible. Although it is recognized that this active mass screening (AMS) strategy has saved thousands of lives [2, 8], it is also recognized that in low-prevalence contexts, such a costly and labor-intensive strategy that detects so few cases, is no longer efficient. Moreover, with decreasing disease prevalence, the population no longer perceives HAT as a threat and shows reluctance toward repeated AMS, leading to a decrease in participation rates [14, 21]. This was clearly the case in the Bonon focus where the last epidemic situation observed in Côte d’Ivoire was contained at the beginning of the 2000s [13, 22, 24]. No HAT cases were detected in 2012 during an AMS; nevertheless, several HAT cases are still regularly diagnosed passively, most of them in the second stage of the disease [15].

In such a context, it is important to reinforce passive surveillance strategies integrating HAT control activities into the general healthcare system and focusing on patients self-presenting for consultation [7, 20, 23, 26]. However, integration of this strategy encounters political and technical challenges partly due to difficulties in diagnosing the disease as well as the capacity of health facilities to cover the population at risk, generally living in remote rural areas far from the main health structures [12, 20]. Moreover, case detection in passive surveillance is first based on clinical symptoms leading to the detection essentially of late stage cases that were probably parasite reservoirs for years before diagnosis and treatment was made [15].

With the objective of reaching HAT elimination in the Bonon focus and based on the hypothesis that subjects living close to HAT patients, and sharing the same environment at risk, have a higher probability of being bitten by infected tsetse flies [9], we implemented a door-to-door strategy in 2012 focused on the immediate vicinities of former HAT patients previously detected in the focus. In this paper, we describe this targeted door-to-door (TDD) strategy and compare the results with those of AMS performed from 2000 to 2012.

## Methods

### Ethics statement

All samples were collected within the framework of medical surveys and epidemiological surveillance activities supervised by the HAT National Control Program (HAT NCP). No samples other than those for routine screening and diagnostic procedures were collected. All participants were informed of the objective of the study in their own language and signed an informed consent form. This study is part of a larger project aimed at improving HAT control by assessing the role of the human reservoir in the emergence and/or re-emergence of HAT, for which approval was obtained from the Ivorian National Ethics Committee, No. 0308/MSLS/CNER-P, January 30, 2012.

### Study site and epidemiological context

**Figure 1.**
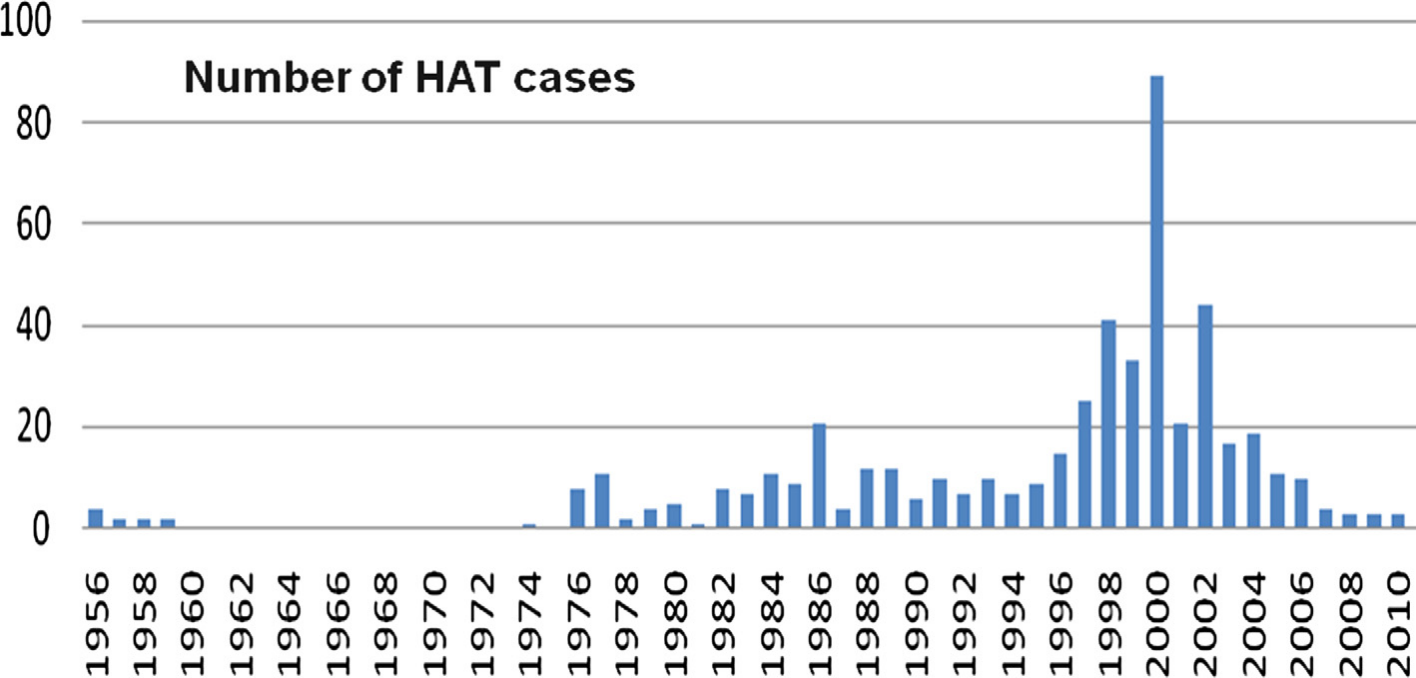
Evolution of the number of HAT cases diagnosed in the Bonon focus from 1956 to 2010.

The Bonon HAT focus is located in the mesophyll forest in the western-central part of Côte d’Ivoire. The disease has long been endemic and the first cases were detected in 1956 [6]. The evolution of the number of HAT cases reported in this focus since the 1950s is well documented based on data supplied by the HAT NCP and partners (see Figure 1). Due to cash crop interests (coffee, cocoa, bananas, etc.) and the associated human settlements, HAT found ideal geographic conditions for its development and Bonon became the most active focus in Côte d’Ivoire at the end of the 1990s [24]. Control efforts conducted between 2000 and 2006 by HAT NCP in collaboration with Institut Pierre Richet (IPR), Institut de Recherche pour le Développement (IRD), and Projet de Recherche Clinique sur la Trypanosomiase (PRCT) managed to contain the epidemic. Since 2007, the disease is hypoendemic with only very few cases passively diagnosed yearly (six cases between 2010 and 2011) [15]. These cases were diagnosed passively (all in an advanced neurological stage of the disease) at the PRCT, the only HAT treatment center in Côte d’Ivoire, located in Daloa, 80 km east of Bonon.

### Classical active mass screening strategy

Specialized mobile teams conducted large-scale screening of all populations considered at risk. Targeted communities then consisted of entire villages or urban neighborhoods. The population at risk was tested using CATT performed on blood (CATT-B) collected by finger prick. For CATT-B-positive persons, blood was collected in heparinized tubes and a twofold plasma dilution series in CATT buffer was tested to assess the end titer, i.e., the highest dilution still positive on plasma (CATT-P). All CATT-P ≥ 1/4 underwent parasitological examinations by direct examination of a lymph node aspirate and/or mini-anion exchange centrifugation technique (mAECT) [18] in blood. From the remaining blood of all CATT-P ≥ 1/4, 500 μL of plasma was collected and kept at −20 °C for subsequent trypanolysis testing in 2012. AMS was performed eight times from 2000 to 2012 (2000, 2002, 2003, 2004, 2007, 2008, 2009, and 2012).

### Targeted door-to-door strategy

Using the database of HAT NCP, IPR, IRD, and PRCT, 109 former HAT cases (FHAT) actively and passively diagnosed since 2000 were referenced. In 2012, two months after the last AMS, a mobile team visited the focus to localize inhabitants and inform them about the aim of the study. The close neighborhood of each localized FHAT, i.e., all subjects living in the same house and subjects living in immediate vicinities and sharing the same daily activities, was then also informed and sensitized about our targeted door-to-door (TDD) strategy. For the subjects who gave their agreement to participate, the same diagnostic procedure as described above was applied. Because most FHAT patients did not participate in post-therapeutic follow-up, we investigated possible treatment failures by clinical, serological, and parasitological means. From the remaining blood of all CATT-P ≥ 1/4 subjects, 500 μL of plasma was collected and kept at −20 °C for subsequent trypanolysis analysis.

New parasitologically confirmed HAT cases (AMS or TDD) were called NHAT and new seropositive cases (CATT-B positive, CATT-P ≥ 1/4 but negative by parasitological tests) were called NSERO.

### Immune trypanolysis test

Plasma samples collected in the field from all NHAT and NSERO individuals were sent to the Centre International de Recherche-Développement sur l’Élevage en Zones Subhumides (CIRDES, Bobo-Dioulasso, Burkina Faso) to be tested by the immune trypanolysis test (TL), highly specific for *T. b. gambiense*, which constitutes a routine surveillance test used nowadays [11]*.* The TL was performed on plasma as previously described [25] with LiTat 1.3, 1.5, and 1.6 variable antigen types (VAT).

### Statistical analysis

The prevalence (number of NHAT/total population surveyed) and seroprevalence (number of NSERO/total population surveyed) data between AMS and TDD were compared using a chi-square test. A Fisher’s exact test was performed when the expected number in a field was ≤5. The level of significance was set at 0.05.

## Results

The number of persons surveyed, as well as prevalence and seroprevalence of AMS and TDD performed in Bonon from 2000 to 2012, is given in Table 1. While the first AMS performed in 2000 took into account the whole Bonon area, because the disease distribution was unknown, the subsequent programs progressively focused on at-risk localities, explaining the variations of the population screened in each survey. Since 2004, the population considered the most at risk (regarding the geographic distribution of previous HAT cases) and surveyed by AMS and TDD is the same. Using AMS, 123 NHAT cases were detected from 2000 to 2004, with prevalence varying from 0.48 to 0.15%. From 2007 to 2009, only six NHAT cases were detected with prevalence less than 0.05%. In 2012, no NHAT cases were detected. Using the TDD in 2012, two months after the last AMS, 1058 individuals were tested in the neighborhood of 72 FHAT cases that were successfully localized and four NHAT cases were detected with 0.4% prevalence, significantly higher (*p* = 0.002) than the AMS performed the same year (Table 1).

**Table 1.** Results of medical surveys in the Bonon focus from 2000 to 2012.

Strategy and year	Total population surveyed	NSERO	Seroprevalence (%)	NHAT	Prevalence (%)
*AMS 2000	15,289	170	1.11	74	0.48
*AMS2002	8284	96	1.16	33	0.4
*AMS2003	1369	25	1.83	2	0.15
AMS2004	6289	40	0.64	14	0.22
AMS 2007	6738	29	0.43	3	0.04
AMS 2008	3276	17	0.52	1	0.03
AMS 2009	4537	28	0.62	2	0.04
AMS 2012	3919	10 (5TL+)	0.25 (0.12)†	0	0††
TDD 2012	1058	10 (7TL+)	0.94 (0.66)†	4	0.4††

† χ^2^ = 9.87, *p* = 0.001.

†† Fisher’s exact test, *p* = 0.001.

* Data already published in [11].

AMS: Active mass screening, TDD: Targeted door-to-door, NSERO: New HAT seropositive individuals, NHAT: New HAT cases, TL+: Positive to the immune trypanolysis test.

From 2003 to 2012, the seroprevalence observed in AMS progressively decreased from nearly 2% to 0.25%, while seroprevalence was 0.94% in 2012 using the TDD. Considering the highly specific TL test now used to differentiate false-seropositive (TL−) and true seropositive (TL+) individuals [8, 9], significantly more SERO TL+ individuals (0.66%) were detected by the TDD in 2012, as compared with AMS (0.12%; *p* ≤ 0.001).

The 72 FHAT cases were tested and no parasites were detected by parasitological investigations. Moreover, no neurological disorder was observed in clinical investigations. Sixty FHATs were still positive on the TL (at least one VAT) and only six remained with CATT-P ≥ ¼.

## Discussion

In 2012, HAT was included in the WHO’s roadmap on neglected tropical diseases with a 2020 target date for elimination as a public health problem, and a 2030 target date for the interruption of disease transmission [8, 22, 26]. It therefore appears more than ever a priority for those working in the field to find the best strategies to achieve this goal. In Bonon, we confirmed that the prevalence of disease has become very low over the past few years and that AMS are no longer efficient to detect infected individuals, even though HAT patients are still occasionally diagnosed passively [15], indicating that transmission of *T. b. gambiense* may still be occurring.

This study describes an alternative active screening strategy consisting of visiting former patients at their home and testing the people living in their close neighborhood using a targeted door-to-door strategy (TDD), and compares it with classical active mass screening (AMS) in the same area. While zero patients were detected by AMS in 2012, four patients were detected by TDD 2 months later with fewer people screened. In addition, TDD also enabled the detection of significantly more NSERO TL+ cases that are believed to be asymptomatic carriers of parasites [3, 12]. The four NHAT patients detected by TDD were clinically healthy and refused to go to the treatment center where lumbar puncture is performed for staging. Although we do not have CSF results, the patients were likely in stage 1 since they had no neurological symptoms. Interestingly, three of them could be tested again in 2015 and they were negative by parasitology and still asymptomatic, but remained positive by serology (data not shown). Such infection processes have already been described previously in this area [12].

One of the reasons why *T. b. gambiense*-infected individuals are missed by AMS is related to the chronic nature of the disease, with severe clinical symptoms usually appearing late during the infection course, and to the existence of asymptomatic individuals able to control their infection over sometimes very long periods [1, 10]. Therefore, a number of infected individuals are either asymptomatic or paucisymptomatic [16] and are not interested in spending time participating in repeated and restrictive medical surveys. Other reasons could be social (attitudes toward the disease and the mobile team, fear of being ill, etc.) and economic (no time to attend the screening because of daily activities). These results also suggest and confirm the lack of interest of the at-risk population regarding repeated AMS in a given area [21]. In contrast, TDD that targets the close neighborhood of FHAT patients is a home-based approach and provides a friendlier environment, inviting inhabitants to participate and gain awareness of the disease. Furthermore, the presence of an FHAT case in the direct neighborhood generally prompts others to accept testing.

The fact that the prevalence of infection is increased in the immediate neighborhood of HAT patients as compared to the general population also provides important information on *T. b. gambiense* transmission in this disease focus. Although the prevalence of disease has been markedly reduced by the control measures implemented since the beginning of the outbreak in 2000, our data suggest that very localized transmission cycles may persist in the environment of FHAT patients. It is important to note here that all FHAT patients were successfully cured and therefore probably did not contribute for a long time to the maintenance of transmission in their environment. Interestingly, the NHAT cases were diagnosed in the immediate neighborhood of FHAT cases detected in 2000 when most cases were diagnosed. This suggests that such localized transmission areas can last for long periods of time. Such hypotheses were already proposed to explain the fact that HAT cases often cluster in particular families [9, 17]. It is also possible that the NHAT cases were infected a long time ago and were not detected by AMS for the reasons described above. In this context, targeted vector control measures, such as the deployment of insecticide-impregnated targets where FHAT or NHAT individuals are exposed to tsetse flies, could significantly reduce the risk of secondary infections and help interrupt transmission. Reducing human-tsetse contacts with impregnated targets was recently shown to be efficient in reducing new infections in the active HAT focus of Boffa in Guinea [4].

Côte d’Ivoire is currently a country where fewer than 10 HAT cases have been detected yearly since 2009 [7], although it is difficult to estimate the true number of cases present because of lack of medical investigation in older HAT foci. In this low-prevalence context, the mass screening approach appears unrealistic to maintain because of low efficiency in detecting the last infected individuals. In such settings, the current strategy is to switch to a passive detection system integrated into the national health structures [22, 26]. An important drawback of the passive approach, however, is that, because it is based on the willingness of HAT patients to present themselves to the local health facilities, these HAT patients are usually diagnosed in the late stage of the disease (increasing the time during which they can act as reservoirs for tsetse flies). The door-to-door approach described here therefore appears to be a useful complementary alternative to maintain targeted active screening across the population. It is therefore very important to carefully geo-localize HAT and SERO TL+ individuals diagnosed during medical surveys and/or by the passive detection system that is likely to be scaled up in the coming years, in order to construct databases on which TDD implementation can rely. In Côte d’Ivoire, we propose the following reactive active case-finding survey strategy, as suggested by WHO [8]: once a HAT case or a SERO TL+ is detected, a small mobile team is sent to screen the immediate neighborhood. Combined with tsetse control measures aimed at reducing human tsetse contacts in their risk environment, as was suggested by [5] in the Forecariah focus (Guinea), we believe that such measures may greatly speed up and ensure a more sustainable elimination process, especially in areas where the disease prevalence is becoming low.
